# Combustion Synthesis of Composition Ferroalloys

**DOI:** 10.3390/ma11112117

**Published:** 2018-10-28

**Authors:** Mansur Ziatdinov, Alexander Zhukov, Vladimir Promakhov

**Affiliations:** Additive Technologies, National Research Tomsk State University, 634050 Tomsk, Russia

**Keywords:** self-propagating high-temperature synthesis (SHS), composite ferroalloys, nitrides, borides, filtration combustion, ferrovanadium nitride

## Abstract

The main objective of this paper is to present results of the research in the development of a specialized self-propagating high-temperature synthesis (SHS) technology for ferroalloy composites, as applied to steelmaking. The problem of creating such a production cycle has been solved by developing a new approach to the practical implementation of self-propagating high-temperature synthesis, as applied to metallurgy. The metallurgical variation of SHS is based on the use of different metallurgic alloys (including waste in the form of dust from ferroalloy production) as basic raw materials in the new process. Here, the process of synthesis by combustion is realized through exothermic exchange reactions. The process produces a composite, based on inorganic compositions with a bond of iron and/or alloy based on iron. It has been shown that in terms of the aggregate state of initial reagents, metallurgical SHS processes are either gasless or gas-absorbing. Combustion regimes significantly differ when realized in practice. To organize the metallurgical SHS process in weakly exothermic systems, different variations of the thermal trimming principle are used. In the present study, self-propagating high-temperature synthesis of ferrovanadium nitride, which is widely used in steel alloying, was investigated. It has been shown that the phase composition of the initial alloy has a profound impact on the regular patterns in ferrovanadium combustion in nitrogen and on the mechanism itself. During the nitriding of σ-(Fe-V), process activation is taking place. The activation is due to the transformation of the intermetallide into an α-solid solution, when the temperature of phase transition is reached (~1200 °C). The composite structure of the products of ferrovanadium is nitriding by the fusion of particles-droplets composed of molten Fe and solid VN.

## 1. Introduction

Currently, steel is the main construction material and it is likely to remain so within the coming decades. Even though the consumption of aluminum, titanium, and other metals (as well as plastics, ceramics, etc.) is increasing, alloys based on iron will be dominating in construction, transportation, power engineering and other industries for a long time to come. The global production of steel is steadily increasing. In 2017 ~1.7 million tons of steel were produced. Over 2 billion tons of ore, ~1.2 billion tons of coal, ~0.2 billion tons of lime, and over 0.06 billion tons of ferroalloys have been used in the production of steel. This places a significant load on the environment. Thanks to metallurgy, environmental problems are already a big challenge to humankind. An increase in the production of steel can be an insurmountable burden for the environment. That is why, improving the mechanical properties of steel is an important approach to sustained economic development, without an increase in the environmental impact. 

The Ultralight Steel Auto Body (ULSAB) is a successful example of this approach, as applied to car manufacturing. Leading steel production companies created new kinds of specialized metals that have allowed for a significant decrease in car weight, while improving car marketability and safety. High physical and mechanical properties have been achieved through the combination of microalloying, refining and new regimes of thermomechanical treatment. Both metals (Al, Ti, Nb, V, etc.) and non-metals (B, P, N, etc.) are used as microalloying elements in novel steels in car manufacturing. 

Inhibition induced by nitrides is another example of how specific metal consumption is decreased through improving metal quality, as applied to transformer steel production. Replacing sulfur with microalloying of nitrogen has allowed for improving the magnetic properties of steel and significantly decreasing transformer weight.

Microalloying is one of the main methods for achieving the required engineering material specifications of steel. Both traditional and relatively new additives, such as nitrogen and boron, are used as microalloying elements. The advantage of nitrogen and boron lies in their abundance, environmental safety and minor concentrations that are required to achieve a positive impact on steel properties. Microalloying puts forward more stringent requirements with respect to the quality of ferroalloys and alloying. Their chemical composition and structure must provide for the high and stable uptake of microalloying elements. For efficient microalloying of steel, the accurate dosage of microalloying elements is required. This can be achieved only by the managed uptake of the elements by the melt, while the alloying consumption remains minimal. 

The chemical composition and structure of modern furnace ferroalloys with nitrogen and boron are well-optimized and stable. Further increasing their quality is possible, by replacing them with ferroalloys having a composite structure. Ferroalloys ensure the required level of steel microalloying at minimum cost. In using composite materials with alloy elements, the microalloying element is represented by fine particles of nitrides, borides, silicides, etc.

Exothermic reactions are the basis of metallurgical processes. Iron smelting in blast furnaces, converter steelmaking, agglomerate production, etc. are pyrometallurgy processes, where the combustion of carbon fuel is used to achieve high temperatures. Such combustion (as well as combustion in heat producing units) can be described as heat producing combustion with the purpose of producing heat. In metallurgy, another combustion type is also widely used, and the metallothermic process is an example. Here, exothermic reactions are used to produce metals and ferrous alloys. 

SHS or combustion synthesis is an alternative to the synthesis of compositions based on oxygen-free components. Synthesis by combustion is unique, in terms of energy efficiency and the possibility of treatment with high temperature and pressure simultaneously, which allows for adding new qualities to the synthesized materials. The attractiveness of SHS lies in its fast rate and the simple design of SHS reactors. SHS technologies have been developed for many industries. However, they have not been applied to metallurgy until now. The main reason behind the rejection of SHS materials in metallurgy is the high cost that arises out of the use of pure metals and non-metals as the raw stock for the powders. Thus, one of the main economic benefits of SHS technology, zero energy costs, is eliminated by the cost of the initial materials. In addition, the current demand of metallurgical production greatly outweighs the currently available production capacities of traditional SHS materials.

Researchers have responded to this challenge with the creation of the so-called metallurgical SHS process, i.e., a synthesis process by combustion, whereby, its products are suitable to be used in steelmaking and blast-furnace ironmaking. A distinct feature of the metallurgical SHS process is the use of iron-based alloys and alloying elements (produced by traditional metallurgical processes) as the main raw stock. The replacement of chemical raw stock (metal and non-metal powders) with metallurgical raw stock (ferroalloys that are cheaper and more available), has allowed for the transition from laboratory-scale production of traditional SHS materials to industrial production for SHS.

**Figure 1:**
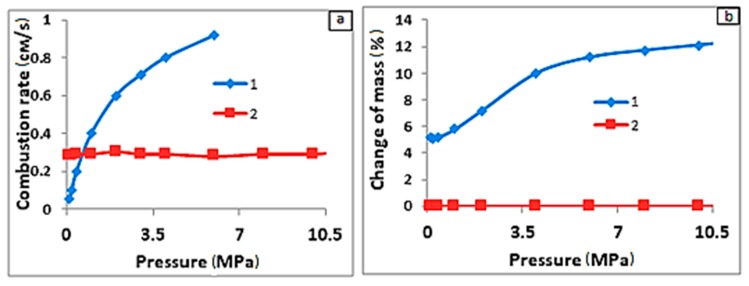
Burning velocity (**a**) and mass change (**b**) as a function of gas pressure for the following systems: (1) FeTi + N_2_, (2) FeB + Ti (Ar) [3].

The traditional SHS process was supported by the heat of direct synthesis reactions [1,2]. The metallurgical alternative uses ferroalloys as the main reagents of initial exothermic stock [3] and the SHS process relies on exothermic exchange reactions (Table 1). Such reactions are close to metallothermic reactions. The main raw stock of the metallurgical SHS process (i.e., ferroalloys) are silicides (FeSi, FeSi_2_, MnSi_2_), intermetallides (VFe, TiFe, Nb_19_Fe_21_), borides (FeB, FeB_n_), etc., and solid solutions (Cr (Fe)), as well as different combinations of these [4]. The SHS process, based on these combinations, yields a composite based on inorganic compositions with a bond of iron and/or alloy based on iron. In terms of the aggregate state of initial reagents, the metallurgical SHS process (as well as the traditional process) can be gasless or gas-absorbing (Table 1). Differences between the combustion regimes of both are provided in Figure 1. In the case of gasless combustion, there is no dependency of the rate and temperature of combustion on the external gas pressure. Here, the mass of the raw stock is equal to the mass of the yielded combustion products. During gas-absorbing combustion (filtration combustion), the mass of the synthesized products exceeds the mass of the raw stock. The increase is due to the uptake of nitrogen by the initial ferrous alloy. Here, a strong dependency of the combustion rate and temperature on the pressure of nitrogen is observed.

The chemical oven is one of the chemical routes of SHS reactions. Here, chemical reactions flow independently of each other, and the heat from a strongly exothermic reaction facilitates the realization of another, not so exothermic, reaction. To organize the SHS process in weakly exothermic mixtures, either external energy is used, or the existing energy is recuperated. External energy can be input as physical or chemical heat. Electrical furnaces are normally used for physical heating for SHS. To increase the exothermic output of SHS raw stock, we can add more chemical heat to it. In alumothermic processes, this technique is used to obtain complex ferroalloys [4].

**Figure 2:**
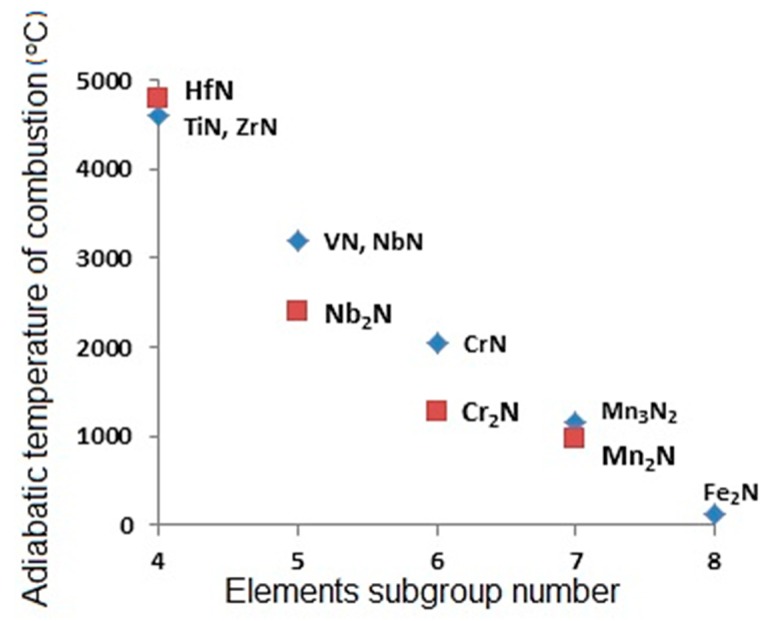
Adiabatic temperature of combustion reactions nitrides [6].

For the first time, the external chemical oven was used for synthesizing intermetallides of Nb-Al and Nb-Ge systems in the gasless combustion regime [5]. For filtration combustion, the chemical oven was suggested for metallurgical SHS process systems in order to obtain complex alloying elements, based on Сr and Mn [6]. In the case of formation of Cr and Mn nitrides, the heat release is much less than the heat release during the synthesis of nitrides of III-V group metals of the Periodic Table (Figure 2). That is why, to increase heat output, (Fe-Cr) and (Fe-Mn) powders are mixed with Al, Ti, V, etc. powders or with alloys of iron and these powders. Here, rather than an external chemical oven, there is an internal chemical oven that is used: the raw stock of the target product and the raw stock of the chemical oven are not spatially divided. Such a technique can be used for producing alloying elements, if the combustion products of the chemical oven are compatible with the composition of the steel to be produced. Nitrides of Al, V and other metal-donors of chemical heat are the same sources of nitrogen for steel as Cr and Mn nitrides of heat acceptor metals. The principle of the internal chemical oven is an efficient method for synthesizing complex alloying elements containing nitrogen. This principle allows for obtaining compositions with different levels of nitrogen concentration, thus, allowing for a wide variation of other components. The new technique of the internal chemical oven was successfully implemented in the realization of a gasless variation of the metallurgical SHS process for obtaining composite alloying elements containing boron [7]. Here, this technique enables the synthesis in mixtures with alloys that have low boron concentration. For the metallurgical SHS process, the external chemical oven is used when the products of the donor mixture combustion are not compatible with the synthesized acceptor materials (Table 2). 

The advantage of N and B as alloying elements lies in the fact that their positive impact on the properties of steel is manifested even at low concentrations [8,9,10]. Notwithstanding, N and B are abundant, and they are environmentally safe. The content of N in steels required by technology specifications may vary from tens of ppm to ~1% N. Currently, high-nitrogen steels are widely used in power engineering, drive engineering, chemical technology, and other industries. High-boron stainless steels are indispensable in nuclear power engineering. Steels with micro-additions of N and B are now widespread.

Nitrogen naturally occurs as gas, therefore, to introduce it into a steel, it must be taken up as part of a solid. Such a material containing nitrogen must be compatible with the steel melt and it must also be processable. That is why nitrided ferroalloys and metals are normally used as sources of nitrogen. Versatility is the advantage of steel nitriding using alloying elements. Such alloying elements can be used in steelmaking, with all types of steel-melting devices at metallurgical plants with different equipment capability. Using alloying elements containing nitrogen, the entire range of steels can be produced; micro-alloyed by nitrogen and with maximum nitrogen content. Metal alloying elements are environmentally safe. That is why nitrogen alloying using metal alloying elements is now the main process technique for steel nitriding. Nitriding is used on Mn, Cr, V and Si alloys.

The realization of composite ferroalloys technology based on the metallurgical SHS process, is now considered in this section, by using the example of ferrovanadium nitride composite.

In isothermal conditions, the mechanism of the interaction of metals with nitrogen is complex and largely differs from the mechanism of nitriding of individual metals. The process of interaction is influenced by multiple factors: the phase and elementary composition of the alloys and the different degree of affinity of alloy components for nitrogen. Nitriding is accompanied by the diffusion stratification of alloys, which leads to a decrease in the process speed. The reaction ability of the alloys is strongly influenced by phase transitions taking place inside the alloys during high-temperature treatment. It can be expected that, in the strongly non-isothermal conditions of the combustion wave, the peculiarities of the alloys-gas interaction may have their impact. Eventually, this can result in different combustion process patterns and mechanisms, as well as differences in the structuration of synthesis products. 

Thus, the purpose of the work is the synthesis of composite ferroalloys by combustion using the example of producing ferrovanadium nitride.

## 2. Materials and Methods

The research into consistent patterns and mechanisms of (Fe-V) combustion in nitrogen was conducted using vanadium electrolyte (99.8% V) and aluminothermic reaction—vanadium alloying —(97.8% V), iron electrolyte (99.98% Fe), and the following industry-grade alloys: FeV 80 (78.8% V), FeV 60 (59.2% V), FeV 50 (52.4% V), FeV 40 (41.6% V). Model alloys (Fe-V) of different composition were produced by melting powders of V electrolyte and Fe in a vacuum furnace. In this research, alloys with the estimated content of 80.0, 70.0, 60.0, 55.0, 48.0, 40.0 and 35.0% V were obtained. All the alloys were single-phase: at 60.0%–80.0% V they were a solid solution based on α-V, and at 35.0%–55.0% V there was a VFe σ-intermetallide. The combustion rate was determined using a video camera. The content of nitrogen in (Fe-V) combustion products was determined on a LECO TCH-600 analyzer. The combustion temperature during vanadium-iron nitrogenation was measured using W/Re 5/20 thermocouples. To determine the stages of nitrogen uptake during the combustion of (Fe-V), the synthesis in a specialized SHS unit that allows for continuous logging of sample weight changes throughout the entire process was conducted.

Tempering (the stopping of the synthesis process) was performed by rapidly cooling the burning samples. To perform layer-by-layer analysis of the tempered alloys, samples from the layers adjacent to the combustion front, from the side with the nitrogenation products (at ~0.5 mm) were taken. To analyze the structure of the combustion products, thin sections were made. The density and porosity were determined by using hydrostatic weighing. The nitrogen pressure during the synthesis was 6 MPA.

## 3. Results and Discussion

**Figure 3:**
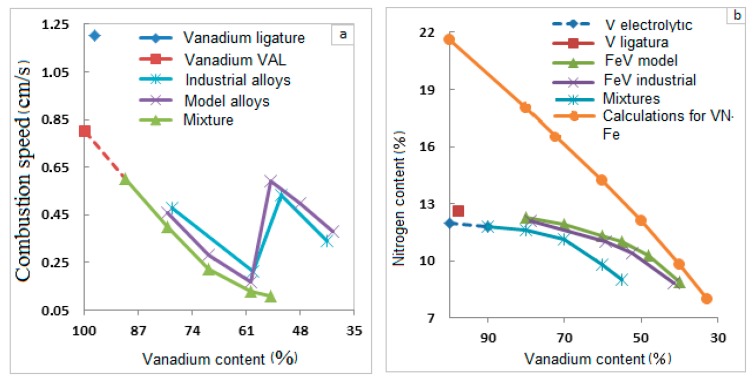
The impact of V concentration on the burning rate and nitrogen concentration in (Fe-V).

In the V-Fe system, the reactions yielding V nitrides are exothermic, hence, lowering the concentration of V in them reduces the possibility of process support by self-sustained combustion. All alloys burn in nitrogen when the concentration of vanadium in them is at least ~40% (Figure 3). The rate of the mixture V and Fe powders is decreasing monotonically as the content of Fe increases, until it reaches its maximum of 45%. Mixtures with a high iron content do not burn. The rate of combustion of (Fe–V) alloys decreases when the concentration of vanadium in them decreases from 80% to 60%. As the content of vanadium in the alloy is further decreased, the rate of combustion increases rapidly. When the content of V changes from 60% V (the model alloy) to 55% V, the rate of combustion increases from 0.15 to 0.58 cm/s. When industrial ferrovanadium compositions are nitrided, the transition from a 59.2% V alloy to a 52.4% V alloy leads to an increase in the combustion rate from 0.21 to 0.52 cm/s.

**Figure 4:**
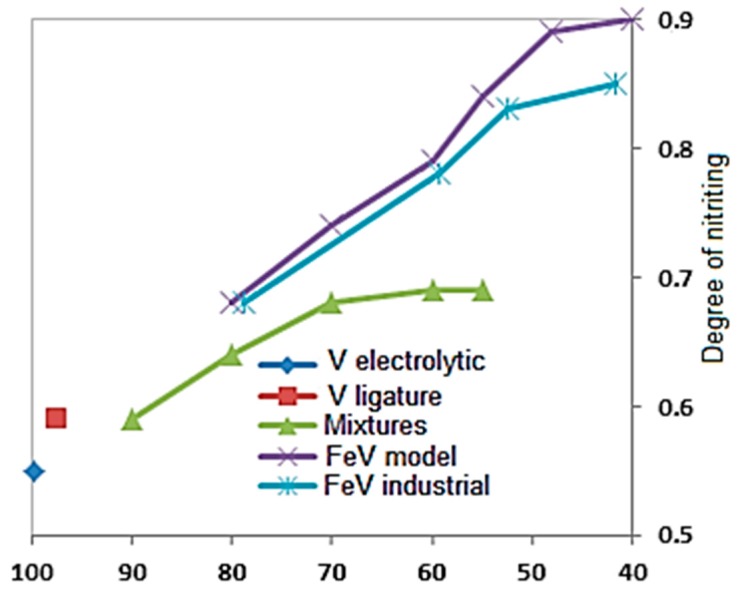
The impact of V concentration on the degree of nitriding of (V-Fe).

Also, the concentration of N in the products of V-Fe system combustion decreases if the iron content is increased (Figure 3b). The content of N in the products of combustion of model- and industrial-grade ferrovanadium differs insignificantly. This content is also very far from the maximum values. The extent of V transformation into the nitride (the degree of nitriding) is a ratio of nitrogen content in the combustion products to the maximum possible nitrogen content in the metal when VN_1.0_ is formed:

(1) $$m=\frac{\mu_{V}M_{N}}{\mu_{N}M_{V}}\times 100\%,$$

where $\mu_{V}$ and $\mu_{N}$ are the atomic masses of V and N, $M_{V}$ and $M_{N}$ are the amounts of V and N in the combustion products. The degree of nitriding increases as the concentration of Fe in the combustion products is increased (Figure 4). 

In the research of synthesis by the combustion of V and Cr nitrides [10,11,12], it has been shown that when the pressure is below 15 MPa, the content of nitrogen in the pores of raw powder stock is significantly lower than the minimal amount that is necessary to sustain the process in the self-propagation regime. In the case of nitriding of ferrovanadium, the above-mentioned minimum pressure depends on the content of Fe in the alloy. To evaluate this pressure, the previous formula for calculating the extent of ferrovanadium transition is used:

(2) $$m=\frac{\mu_{V}\rho_{N}V_{N}}{\mu_{N}\rho_{V}\left(V-V_{N}\right)}\cdot \left(\rho_{Fe}-\rho_{V}+\frac{\rho_{V}}{k}\right)\cdot p,$$

where: 

$\rho_{Fe}$ and $\rho_{V}$—the densities of iron and vanadium;

$\rho_{N}$—the density of nitrogen at 0.1 MPa;

$\mu_{Fe}$ and $\mu_{V}$—the atomic masses of iron and vanadium;

*V* and $V_{N}$—the volume of the sample and the volume of nitrogen in it;

*k*—the concentration of vanadium in the alloy;

p—nitrogen pressure.

Maximum pressure used for (Fe-V) nitriding, 14.0 MPa. If the porosity of the V raw stock is V ~50%, then there is enough in the pores to turn only 11% V into vanadium nitrite, VN_1.0_. In the case of transition to (Fe-V), the amount of V transformed into VN_1.0_ increases to 13% for the 80% V alloy, and it increases to 18% for the 50% V alloy. The calculation of Т_ad_ shows that in such conditions maximum temperature can only reach 400–450 °C. The measured combustion temperatures of V and (Fe-V) were 1420–2060 °C. 

**Figure 5:**
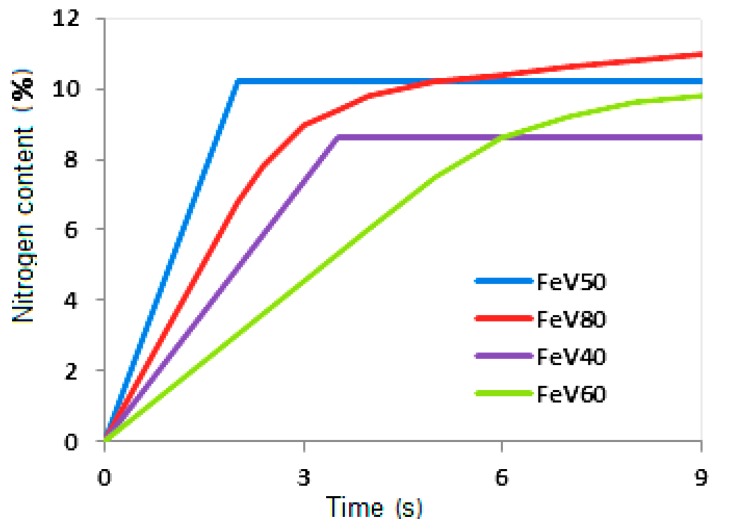
Gravimetric curves of ferrovanadium nitriding.

Figure 4 and Figure 5 demonstrate that the content of nitrogen that is uptaken by the samples is much higher than the amount of nitrogen that can be taken from the pores. Thus, most nitrogen is delivered into the reaction zone from the burning sample environment by means of filtration through its porous part. Such filtration occurs and is sustained by continuous nitrogen uptaking by the zone of chemical reaction, and the pressure imbalance that is created as a result of it.

When (V-Fe) alloys are nitrided, the uptake of nitrogen may take place stage by stage. For the stage-by-stage regime, it is required that in the combustion wave, the degree of transformation of nitrided metal (V) into the nitride must not be maximal, i.e., some fuel must remain for volumetric combustion. Here, the nitrogen containing pre-product that has been formed behind the combustion front must retain a high permeability (porosity), so that nitrogen from the environment of the sample could be freely fed inside to be uptaken as nitrides.

Figure 5 shows gravimetric curves obtained using such a unit for industrial alloys. The curves demonstrate that, in the course of the nitriding of the ferrovanadium alloy within the limits of the existence of the α-phase (V > 55%), a two-stage nitrogen absorption regime is realized. During the first stage, in the layer-by-layer combustion regime, ~75% N is absorbed, and the remaining nitrogen is uptaken by α-(Fe-V) in the volumetric afterburning regime. Within the conditions of the σ–phase existence (35%–55% V), the alloy is nitrided in the single-phase regime. All nitrogen is absorbed during the phase of layer-by-layer combustion.

**Figure 6:**
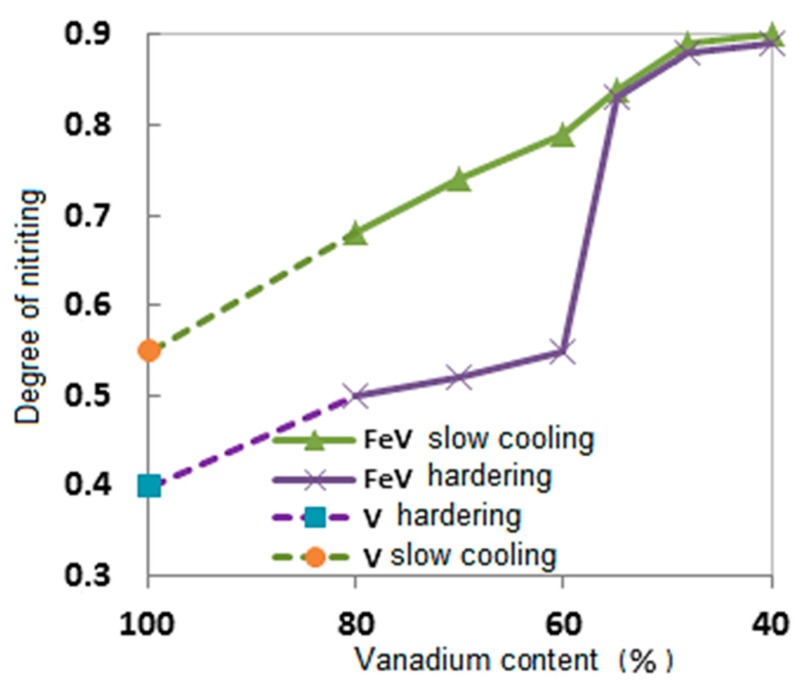
The impact of vanadium concentration on the extent of nitriding of (Fe-V).

The results of layer-by-layer chemical analysis of the tempered samples are the confirmation of the existence of a different mechanism of α- and σ-(Fe-V) nitriding. The obtained results are presented in Figure 6. Curve 1—nitrogen content in the products of alloy combustion during slow sample cooling. Curve 2—in tempered samples. For V and α-(Fe-V) with 60.0%–80.0% V, the concentration of N in the tempered samples is lower than the N concentration in slowly cooling samples, and for σ-(Fe-V) (40.0%–55.0% V) these concentrations are the same. Thus, during the combustion of σ-(Fe-V), nitrogen is uptaken into the product within one phase, directly in the zone of layer-by-layer combustion. In the case of α-(Fe-V) combustion, nitrogen absorption is two-phase.

**Figure 7:**
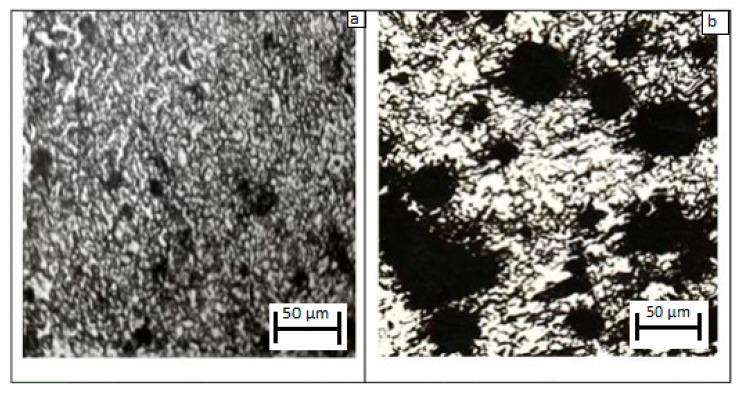
Typical microstructure of nitriding (Fe-V), (**a**) σ-alloy; (**b**) α-alloy.

The analysis of the structure and appearance of the combustion products has shown that σ-(Fe-V) samples after combustion have a molten structure with a porosity of less than 10%. These pores are isolated micropores that are evenly distributed throughout the volume (Figure 7). There are no conditions for an exhaustive reaction in this case. In the case of α-(Fe-V) nitriding, the samples after combustion have a porosity that differs insignificantly from the initial porosity. In this case, a significant amount of nitrogen is absorbed during full nitriding. 

**Figure 8:**
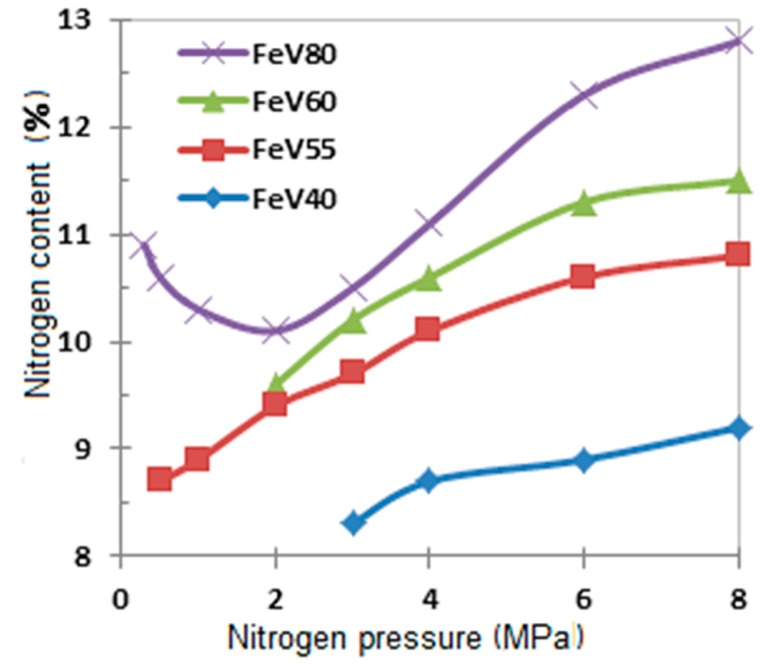
The impact of nitrogen pressure on the extent of ferrovanadium nitriding.

The nitriding of (Fe-V) takes place in the filtration combustion regime. The distinct features of such a SHS process are the dependency of combustion and composition of reaction products on the pressure of nitrogen, fineness of the initial powder, and permeability of the raw stock powder. It was discovered during this research, that the following factors lead to increased combustion rate: increased nitrogen pressure, decreased particle size in the initial alloy, and increased porosity of the samples. The amount of nitrogen in the combustion products is higher in the case of high nitrogen pressure (Figure 8).

**Figure 9:**
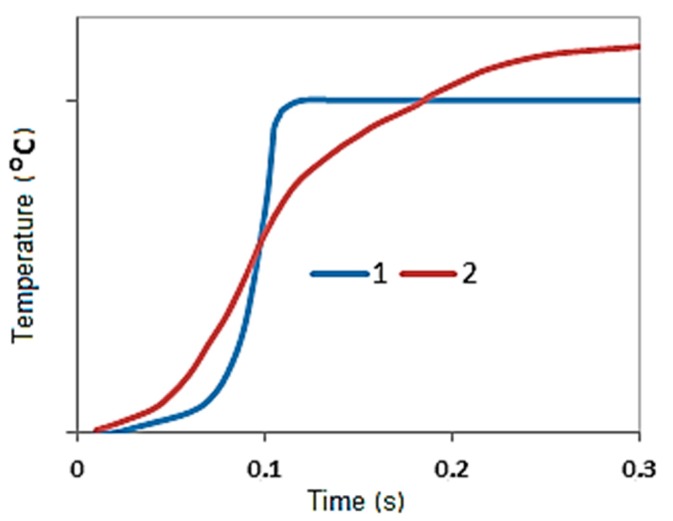
Typical temperature profiles of σ-(Fe-V) (**1**) and α-(Fe-V) (**2**).

Figure 9 shows typical temperature profiles for α- and σ-(Fe-V). Depending on the process conditions, maximal combustion temperatures that are measured during the experiment may vary widely: for FeV80 alloy, from 1780 to 2060 °C; for FeV60, from 1630 to 1830 °C; for FeV50, from 1480 to 1560 °C and for FeV40, from 1420 to 1490 °С. Maximal temperatures correlate with the content of nitrogen in the product: the more nitrogen that is absorbed by the alloy, the higher the temperature that is observed during the combustion.

**Figure 10:**
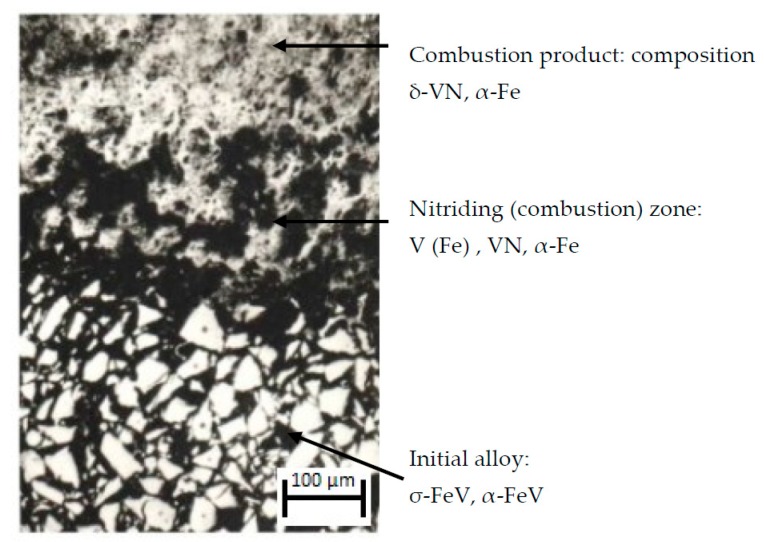
The structure of the σ-FeV combustion zone, particle size ≤ 50 µm.

**Figure 11:**
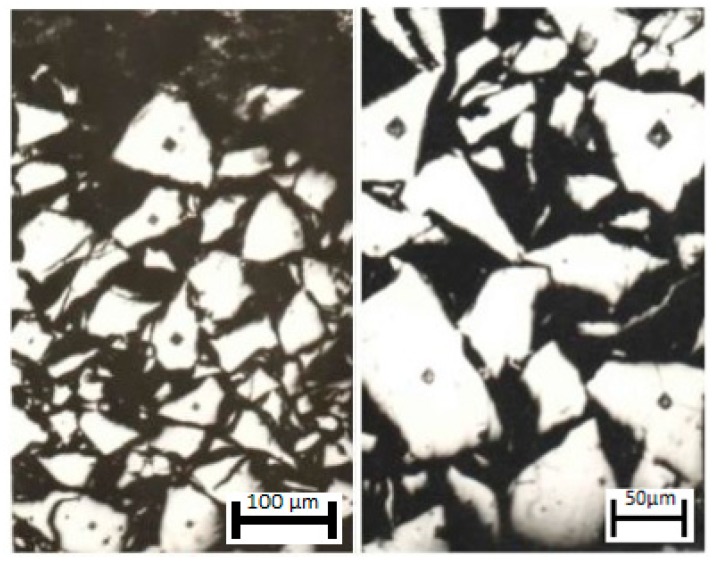
The structure of the σ-FeV combustion zone, particle size ≤ 50 µm.

Layer-by-layer metallographic and X-ray diffraction analysis of the samples, with the combustion front stopped by tempering, has shown that in the course of σ-(Fe-V) nitriding, process activation takes place. The activation is associated with the transformation of the σ-intermetallide into an α-solid solution when the phase transition temperature is reached (~1200 °C for stoichiometric σ-(Fe V)). Now, the mechanism of the formation of two-phase structure of the combusted σ-(Fe V) and gradual transformation of the initial alloy into a composite is traced. This is done by analyzing the zone of combustion of the tempered sample that is obtained from the thin section (σ-alloy FeV50, 50–100 µm) (Figure 10). Here, three zones with different microstructures are clearly visible. The initial ferrovanadium appears to have many light-colored particles with regular faceted patterns. The final alloy is a dense composition consisting of δ-VN and α-Fe. Between these two areas, there is a narrow zone (~0.1–0.3 mm), that is a network of molten particles. Figure 11 shows the structure of multiple layers of initial powder particles adjacent to the transition area, at a greater magnification. It is visible that the microhardness of particles, that are closest to the transitional zone, is lower than the microhardness of the particles from more remote areas. The microhardness of the particles located farther from the transition zone is 1300–1500 units, and it is the same as the microhardness of the initial σ-(Fe-V), and the microhardness of particles in the layers that are closest to the transition zone of the layers decreases to 300–500 units. Such microhardness is close to that of α-(Fe-V). When the microhardness changes, the particles retain their fragmentary shape, which is clearly visible from Figure 11, that shows adjacent particles with different microhardnesses. The microhardness of the particles that are in the immediate vicinity of the transition layer is ~400 units. In the layer adjacent to the combustion front, microhardness increases to ~1300 units. The identical appearance of particles without signs of melting indicates that changes in the microhardness of σ-(Fe-V) take place in the solid state: when heated to over ~1200 °C σ-phase transforms into an α-solid solution. According to [13], this transformation takes place at a high rate. In [14] the microhardness of an α-solid solution that is hardened by tempering (an alloy with 40%–50% V at 1300 °C) was 300–400 units. Thus, in the heated zone when the temperature of phase transition is reached, a σ → α transition takes place. This transition is discovered through a change in the microhardness. This is confirmed by the results of the layer-by-layer X-ray diffraction analysis of the burning zone in the tempered samples. The powders that were ground off in parallel to the combustion front were analyzed. Before combustion, the powder is in σ-phase. In the area adjacent to the combustion zone, a very thin layer of particles is found. When analyzed by X-ray diffraction, this layer looks similar to an α-hard V (Fe) solution. In deeper layers, apart from the solid solution, δ-VN and α-Fe can be observed with an amount that quickly increases. The final product is two-phase and consists of δ-VN and α-Fe.

**Figure 12:**
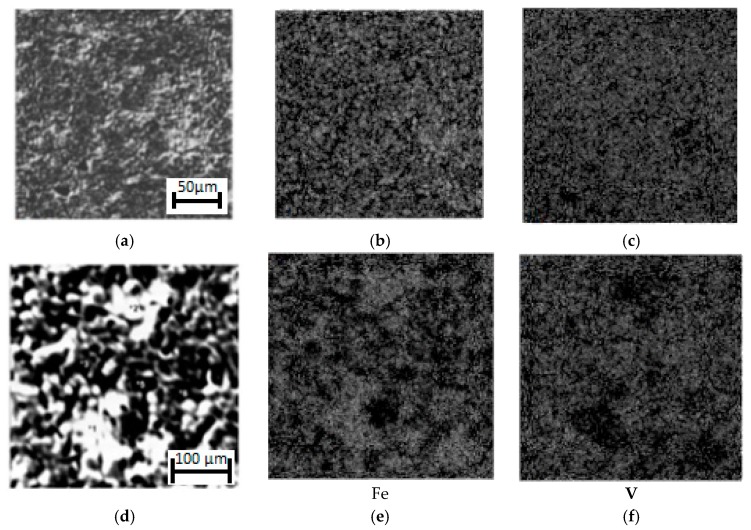
The microanalysis of σ-Fe-V combustion products. (**a**,**d**): structure of materials. (**b**,**e**): Fe; (**c**,**f**): V.

Figure 12 shows the microstructure and microanalysis of nitrided σ-FeV50 at different magnification. The structure of σ-(Fe-V) combustion products is a typically composite one. The size of vanadium nitride nodules does not exceed~0.1 mm. The size of iron layers is of the same order of magnitude. Table 3 shows the results of the microanalysis of nitrided FeV50 alloy, and those results confirm the composite structure: the areas that are rich in iron are interspersed with areas where vanadium is predominant. As a result, of σ-FeV nitriding, dense material with cast structure is formed, and its porosity does not exceed 10%. Interestingly, such a dense product is formed regardless of the density of the initial raw stock. When the initial porosity varies within the range of 35%–55%, the porosity of the product always remains in the range of 3%–10%. Thus, the volume of the combusted samples becomes approximately two times smaller than the initial volume. 

The combustion temperature of σ-(Fe-V) in the entire range of initial parameters (vanadium content in the alloy, pressure of nitrogen, powder fineness, raw stock porosity and concentration of impurities) was in the temperature range of 1420–1550 °C. Therefore, in the case of model alloys, the combustion temperature corresponds to the melting temperature of Fe and iron alloys with minor additions of V and N, and in the case of industrial alloys, it corresponds to the melting temperatures of Al, Si, Mn, and C. At these temperatures, vanadium mononitride (VN) is in solid state, as its melting temperature is 2260–2340 °C for VN_0.7_-VN_0.98_. Thus, a narrow solid-liquid layer is formed in the combustion wave. This layer consists of an iron melt or an alloy of iron and crystalline vanadium nitride.

As opposed to σ-(Fe-V), the products of α-(Fe-V) combustion always contain multiple vanadium-nitrogen compositions. In this regard, α-(Fe-V) is similar to metallic vanadium [10]. During vanadium nitriding, it was discovered that the nitrogen pressure has the greatest impact on the phase composition of the combustion products. Depending on the nitrogen pressure, in the synthesis products the already-known β- and δ-nitrides were found, and a previously unknown γ-phase and a debatable β′-phase were also found. In the course of α-(Fe-V) nitriding, the phase composition of the combustion products also strongly depends on the pressure. Their qualitative composition is virtually the same in the entire range of applied pressure (0.2–14.0 MPa). When X-ray diffraction was used to analyze all the combusted samples, then not only α-Fe but also β-, γ- and δ-nitrides were discovered. When the pressure (and, therefore, the content of nitrogen) is changed, the ratio of the above phases changes. In the interval of 0.5–4.0 MPa, the β-phase is the predominant nitride in the combustion products. As the pressure increases, the amount of this phase decreases, while that of the δ-phase increases. At 10–14 MPa, the combustion products predominantly consist of γ- and δ- phases. Similar to the phase composition of the combustion products, the structure of the final α-(Fe-V) material significantly differs from the structure of the combusted σ-(Fe-V). The main difference lies in the fact that if the structure of the products of the σ-alloy combustion in the stationary combustion zone is virtually the same, then the structure of the final α-alloy material strongly depends on the pressure (which is similar to vanadium). Another difference between the structure of burned α-(Fe-V) and that of σ-(Fe-V) is its cross-sectional heterogeneity, which is especially prominent at high pressure. 

## 4. Conclusions

The conducted research has shown that synthesis by combustion in V-N_2_ and FeV-N_2_ systems is possible in a wide range of nitrogen pressure values, fineness of the initial powder, and the size and porosity of the samples. In the researched pressure range (0.1–15 MPa), nitriding of both vanadium and ferrovanadium takes place in the filtration regime. Higher pressure promotes both the combustion rate and ferrovanadium nitriding. Qualitative differences were found in the consistent patterns and the mechanism of ferrovanadium combustion with different phase composition. It has been shown that the high rate of σ-FeV combustion is due to a σ→α phase transition taking place in the zone of combustion wave heating. Phase transition also explains the high degree of σ-alloy nitriding. Explosive nitriding, because of a σ→α reaction, also promotes the formation of a two-phase α-Fe–δ-VN solid-liquid alloy and the subsequent accelerated compaction with the emergence of a virtually poreless composite structure. The mechanism of accelerated compaction is the reason there is no final (exhaustive) reaction stage in the case of σ-ferrovanadium nitriding.

## Figures and Tables

**Table 1:** Exothermic SHS reactions.

Traditional SHS Reactions	Metallurgical SHS Reactions
**Gasless Synthesis**	
Ti + B → TiB_2_ Ni + Al → NiAl Mo + Si → MoSi_2_ Ti + Ni + C → TiC + NiTi Ti + W + C → TiC + WC	(Fe-B) + Ti → TiB_2_ + Fe FeTi + C → TiC + Fe FeS + Mn → MnS + Fe FeSi_2_ + FeTi → Ti_5_Si_3_ + Fe (Fe-Ti) + B_4_C → TiB_2_ + TiC + Fe
**Gas-absorbing Synthesis**	
Zr + N_2_ → ZrN Ta + C+ N_2_ → TaCN Zr + Nb + C + N_2_ → ZrNbCN	FeV + N_2_ → VN + Fe FeSi_2_ + N_2_ → Si_3_N_4_ + Fe (Fe-Сr) + N_2_ → CrN + Fe

**Table 2:** Examples of synthesis by combustion in the chemical furnace.

Process	Internal Chemical Oven	External Chemical Oven
Traditional SHS process	(Ti + C) + (W + C) → TiC + WC	[Ti + B → TiB_2_] + [B + C →B_4_C] [Ti + C → TiC] + [Nb + C → NbC]
Metallurgical SHS process	[(Fe-Ti) + Si] + [(Fe-Ti) + (Fe-B)] → TiB_2_ + Ti_5_Si_3_ + Fe (Fe-Ti) + (Fe-Mn) + N_2_ →TiN + Mn_4_N + Fe	[(Fe-Ti) +C → TiC + Fe] + [(Fe-Ti) + (Fe-B) → TiB_2_ + Fe] [(Fe-Si) + N_2_ → S_3_N_4_ + Fe] + [(Fe-Cr) + N_2_ → CrN + Fe]

Note: Compositions of the chemical oven are highlighted.

**Table 3:** The results of the microanalysis of nitrided FeV50.

Element	1	2	3	4	5	6
V	18.447	14.021	61.628	7.599	75.286	75.195
Fe	67.417	73.809	20.428	80.278	3.489	3.353
N	14.136	12.170	17.944	12.122	21.225	21.453
Phases	Fe, VN	Fe, VN	VN, Fe	Fe, VN	VN, Fe	VN, Fe
